# Neurological Diseases: A Molecular Genetic Perspective

**DOI:** 10.3390/ijms241310894

**Published:** 2023-06-30

**Authors:** Maryam Ardalan

**Affiliations:** 1 Department of Physiology, Institute of Neuroscience and Physiology, Sahlgrenska Academy, University of Gothenburg, 40530 Gothenburg, Sweden; maryam.ardalan@cfin.au.dk; 2 Department of Clinical Medicine, Translational Neuropsychiatry Unit, Aarhus University, 8240 Aarhus, Denmark

Neurological disorders (which include a broad spectrum of central nervous system diseases from children to old people) remain among the most compelling illnesses known to humankind. From histopathological postmortem examinations of human brain tissue or animal models, there is clear evidence showing that neurological disorders have resulted in microstructural remodeling, microvascular dysfunction, and metabolic disturbances. In recent years, progress in both basic and translational research has helped to elucidate details on the molecular signaling and genetic regulation of neurological disorders as potential molecular bases. However, as a detailed histological analysis is not a possible approach in clinical settings, there is a crucial need to improve molecular approaches. Accordingly, we arranged a Special Issue that focused on the advances in our understanding of neurological disorders by considering genetics, molecular biology, cell biology, neuroimmunology, pharmacology, and therapeutic approaches. This new Special Issue, entitled “Neurological Diseases: A Molecular Genetic Perspective”, in the *International Journal of Molecular Sciences*, includes a total of seven contributions (five original articles, one review, and one case report providing new information on the role of genetic in neurological disorders) which could help to develop novel diagnostic/therapeutic targets. The following is a brief overview of the articles published in this Special Issue.

X-linked adrenoleukodystrophy (ALD) is a condition that causes white matter damage in the adrenal cortex and the nervous system. It is caused by pathologic mutations in the gene that encodes the ATP-binding cassette transporter type D, member 1 (ABCD1). Dohr et al. reported two distinct single nucleotide deletions in the ABCD1 gene: c.253delC [p.Arg85Glyfs*18] in exon 1, leading to both cerebral ALD and a progressive adrenomyeloneuropathy (AMN) phenotype in one family, and c.1275delA [p.Phe426Leufs*15] in exon 4, leading to AMN and primary adrenal insufficiency in other family. In the latter variant, PBMCs lack the ABCD1 protein and have reduced ABCD1 mRNA expression. There was no genotype–phenotype correlation in X-ALD, which was in agreement with the difference in mRNA and protein expression in the index patient and heterozygous carriers. Based on the results of this study, early recognition and diagnosis are key to improving outcomes and preventing premature deaths [1].

Free radicals damage cells by causing oxidative stress. Oxidative stress is effectively countered by antioxidants by quenching free radicals. In addition to being antioxidants, carotenoids are also anti-inflammatory, according to a growing body of research. The carotenoids are cleaved by two homologous enzymes: β-carotene oxygenase 1 (BCO1) and β-carotene oxygenase 2 (BCO2). Janusz Strychalski et al. studied rabbit adipose tissue and brain to investigate the effects of the BCO2 genotype and marigold flower extract supplementation on the expression of BCO1, BCO2, LRAT, and TTPA genes. They found that a diet high in lutein may increase BCO1, LRAT, and APTT gene expression in adipose tissue and BCO2 gene expression in the brain of BCO2 del/del rabbits. They showed that both the adipose tissue and brain of rabbits contained higher levels of carotenoids and tocopherol. As a result of these findings, we can gain a deeper understanding of carotenoid metabolism in rabbits and other animals, which may help therapeutic targets based on carotenoids to be developed [2]. For example, carotenoids are less common in patients with schizophrenia [3].

One of the important neurological disorders is epileptic seizure, affecting 1% of the world’s population. Individualizing epilepsy treatment based on etiology and seizure severity could be made possible through biomarkers. Elzbieta Bronisz et al. hypothesized that serum levels of Matrix metalloproteinases (MMPs), including MMP-9, MMP-2, TIMP-1, TIMP-2, S100B, CCL-2, ICAM-1, P-selectin, and TSP-2, as the proteins associated with the blood–brain barrier, could work as possible biomarkers for seizure prediction. They measured the serum levels of these proteins in the serum of 49 patients with epilepsy who were seizure-free for a minimum of 7 days and also 12 months later. They found that serum levels of MMP-2, MMP-9, TIMP-1, CCL-2, and P-selectin were significantly different between the two time points. They concluded that serum levels of MMP-2, MMP-9, and CCL-2 can be considered biomarkers of seizure prediction [4].

The other neurological disorder is a neurodegenerative disease known as amyotrophic lateral sclerosis (ALS), which is characterized by the death of motor neurons (MNs) within the spinal cord, brain stem, and motor cortex. A transcriptional alteration hypothesis was proposed by Banaja P.Dash for stratifying subtypes of ALS caused by different genetic mutations. For the purpose of identifying key pathways underlying the familial forms of ALS (fALS), high-throughput RNA sequencing was performed on motor neurons (MNs) derived from SOD1- and TARDBP-mutant ALS patients and healthy controls. When investigating new therapeutic approaches for genetic ALS and other neurodegenerative diseases, targeting gene-specific alterations could be the most effective approach [5].

One of the important mechanisms underlying neurological disorders is the impartment of synaptic plasticity. Arc/Arg3.1 (activity-regulated cytoskeleton-associated protein (ARC)) was associated with a high level of expression in neuronal dendritic spines. Learning behavior was found to rapidly induce ARC mRNA and protein levels [6]. Wang and colleagues used CRISPR/Cas9-mediated gene editing to generate an ARC-knockout HEK293 cell line and identify differentially expressed genes and proteins. According to their findings, the ARC regulates gene expression in the extracellular matrix, synaptic membrane, and heat shock protein family. They provided new evidence for the mechanisms that underpin ARC’s effects and molecular pathways involved in schizophrenia pathophysiology using transcriptomic and proteomic profiles of ARC-KO HEK293 cells [7].

Parkinson’s disease (PD) is a challenging neurodegenerative disorder in terms of treatment, which has been estimated to affect approximately 12.9 million people by 2040. According to Keelan Jagaran et al.’s review, the purpose of LNPs is to be efficient therapeutic nanodelivery vehicles for treating Parkinson’s disease (PD). Indeed, RNA interference (RNAi) plays a crucial role in silencing genetic mutations associated with PD, such as LRRK2, PARK7, PINK1, PRKN, and SNCA. It is imperative, however, to increase basic research focused on novel formulations directed to the brain in order to address the lack of studies using LNP formulations [8].

Ambra Butera et al. described a case of a child with a paternally inherited truncating variant of the *PHF21A* gene. A gene named PHF21A (PHD finger protein 21A) encodes a protein called BHC80 which is predominantly expressed in fetal brains and is involved in the regulation of several neuronal genes. Patients with this gene mutation suffer from different clinical symptoms, such as epilepsy, hypotonia, and dysmorphic features. Ambra indicated that the EMG analysis should be part of the clinical diagnostic protocol of these patients because the PHF21A gene is abundantly expressed in skeletal muscles as well. As a result of the paternal mosaicism described in this report, the importance of genetic counseling can be further emphasized, which will provide a more accurate assessment of the risk of transmission in future pregnancies [9].

This Special Issue aims to attract clinicians and researchers interested in improving the current understanding of neurological diseases where genetic mutations are frequent. Thanks to authors’ and reviewers’ efforts, this Special Issue has consisted of high-quality articles that are useful to both preclinical and clinical researchers.
